# Mood after stroke: a case control study of biochemical, neuro-imaging and socio-economic risk factors for major depression in stroke survivors

**DOI:** 10.1186/1471-2377-10-125

**Published:** 2010-12-30

**Authors:** Kausik Chatterjee, Susan Fall, David Barer

**Affiliations:** 1 Department of Stroke Medicine, Countess of Chester Hospital, Chester, CH2 1UL, UK; 2 Stroke Research Team, Queen Elizabeth Hospital, Sheriff Hill, Gateshead, NE9 6SX, UK

## Abstract

**Background:**

Though vascular factors may be important in the aetiology of late-life depression, it is not clear whether they have a major effect on the risk of depression after a stroke. We investigated the relationship between physiological, biochemical, neuro-imaging and socio-economic factors and late-phase post-stroke depression in a cross-sectional case-control study.

**Methods:**

People living at home at least 9 months after a stroke were interviewed using a structured proforma. Depression was diagnosed according to DSM-IV criteria, together with a Montgomery Asberg (MADRS) score >17. Stroke survivors of similar age and functional status but without symptoms of, or recent treatment for, depression and with MADRS score <7, were recruited as controls.

**Results:**

Stroke survivors with depression were more likely than controls to have been smokers, to have had hypertension or peripheral arterial disease, and to have had more than one stroke or multiple discrete brainscan lesions. In univariate analysis they had significantly higher blood pressure, lower Mini-Mental State (MMSE) scores, higher serum homocysteine and lower folate levels, as well as more extensive white matter and basal ganglia changes on brainscan. In logistic regression, previous hypertension (OR 3.4), peripheral vascular disease (OR 4.7), number of strokes (OR 2), MMSE score (OR 0.76) and basal ganglia changes (OR 2.2), were independently associated with depression.

**Conclusion:**

These results suggest that patients with hypertension, hyperhomocysteinaemia and other factors associated with cerebral small vessel disease, may be more susceptible to post-stroke depression. Future intervention trials should focus on such high risk groups.

## Background

Although depression is known to be common after a stroke, consistent risk factors are hard to identify from the literature and longitudinal studies suggest that correlates may change with time [1]. Little attention has been paid to biological factors, in contrast with studies of depression in the general elderly population, where high blood pressure [2], diabetes [3], coronary artery disease [4] and other vascular factors [5] have been found to be important.

The longstanding controversy over the relationship between the location of the stroke lesion, and the risk of subsequent depression [6] has perhaps diverted attention away from other important neuro-imaging findings, such as the presence of 'silent' infarcts, diffuse white matter changes, cortical and central atrophy, some of which are associated with late-life depression. The Mood After Stroke study set out to examine the role of vascular and other risk factors and 'chronic' neuro-imaging changes (rather than focusing on the location of the acute stroke lesion) in well defined, clinically confirmed cases of depression several months after a stroke, using a case-control design with group frequency matching.

## Methods

### Patients

Patients living in the community, over 9 months after a stroke (WHO clinical definition, confirmed by a stroke physician), without severe cognitive or communication impairment, were initially screened by post using the 12-item version of the General Health Questionnaire (GHQ12) [7]. 'Potential cases of depression' and possible controls were approached. From the postal information, they were provisionally matched according to age group and current functional status using the 20-point Barthel Index [8] of activities of daily living (ADL), divided into four strata (< 14, 14-18 and 19-20), aiming to find two possible controls for each potential case in each stratum.

### Interview: Neuro-psychiatric Assessment and Case-Control categorisation

Those who consented were interviewed, using a structured proforma, by an investigator (KC) without prior knowledge of the postal questionnaire responses. As well as a standardised neuro-psychiatric examination for DSM-IV [9] classification and Montgomery Asberg Depression Rating Scale (MADRS) [10], the GHQ12 [7], Dartmouth COOP chart [11] and 'Yale' depression question [12] were administered. Cognitive function was assessed using the Mini-Mental State Examination (MMSE) [13].

During the interview, the observations and responses required for the MADRS [10] were noted, and the Diagnostic and Statistical Manual (DSM-IV) criteria [9] for major depression were applied. The final case-control categorization was made at the time of interview.

**Cases **were stroke survivors who satisfied DSM-IV criteria for major depression **and **had MADRS scores >17 [14].

**Control subjects **were stroke survivors who

1 Did **not **fulfil DSM-IV criteria for major depression

2 Did **not **have any minor depressive symptoms at the time of the interview

3 Had **not **been treated for depression within the previous 6 months

4 Had MADRS score ≤6.

### Other Assessments

The Barthel ADL Index [8] was used to assess subjects' self-care ability and the 66-point Frenchay Activities Index (FAI) [15] for instrumental, outdoor and social activities. Self-reported functional status before the index stroke was assessed using the modified Rankin score [16] with specific questions on pre-stroke mobility and continence. Socio-economic factors noted included the level of formal education, socio-economic class (based on self-reported former occupation), council tax band (via postcode) and whether or not they lived alone.

Subjects were asked whether they had been diagnosed with diabetes, hypertension, atrial fibrillation, ischaemic heart disease, cardiac failure or peripheral arterial disease (PAD). Information was later checked against hospital case notes.

Two sitting blood pressure readings were taken at different points during the interview using a calibrated, standard, manual mercury sphygmomanometer (intraclass correlation coefficient between the two readings: 0.9 for systolic and 0.88 for diastolic pressure). Those with an irregular pulse had an electrocardiogram (ECG) performed, unless arrhythmia had previously been documented. If the pulse was regular, ECGs from the casenotes were checked.

A blood sample was collected for routine blood count and biochemical tests, C-reactive protein (CRP), total & HDL cholesterol, triglyceride, serum folate and homocysteine levels. Samples were identified by unique study number only and transported on ice where necessary. Serum folate and homocysteine were only measured in 72 control subjects as 14 samples were lost in the laboratory.

### Brain Imaging

Computed Tomography (CT) brain scans, performed routinely at the time of the 'index' acute stroke, were assessed for chronic changes using a structured proforma. The rating scale for white matter changes (WMC) was based on that devised by the European Task Force [17]. All scans were performed on the same GE Medical Pro-speed scanner, with 5 mm cuts for the posterior fossa and 10 mm for the remaining brain. Subjects' identities were concealed and replaced by a unique identification number.

For inter-rater reliability analysis, 40 randomly selected CT brain scans of study subjects were reported blind by two radiologists and three stroke physicians, using the same proforma. For the main analysis, all available scans were reported separately by two investigators, using the same proforma, and consensus ratings were then agreed.

### Further data collection

Details of the index stroke, including Oxfordshire Community Stroke Project (OCSP) clinical subtype [18] and presence or absence of aphasia in the initial stages, were collected from the hospital case-notes and the Gateshead Stroke Register. The likely location of the acute stroke lesion was determined clinically and classified as right or left hemisphere (including thalamus) or brainstem/cerebellum.

### Statistical analysis

We used a frequency matching procedure to minimize group differences in age and current functional status (Barthel ADL Index).

Cases and controls were initially compared on single variables using one-way analysis of variance. Where appropriate, logarithmic transforms of continuous variables were performed and geometric means compared. Mann-Whitney U test was used for non-interval scale or non-normalisable data, and chi-squared or Fisher's exact tests for comparing proportions.

Those variables showing significant effects in univariate analysis were included in logistic regression models, to minimise confounding and examine their independent contributions.

Inter-rater reliability for assessing brainscans was measured using intraclass correlation coefficients (ICC) for scalable data and Cohen's kappa for categorical items. Statistical analysis was performed with SPSS-11 and EpiInfo-6.

The Local Research Ethics Committees approved this study.

## Results

**Figure 1:**
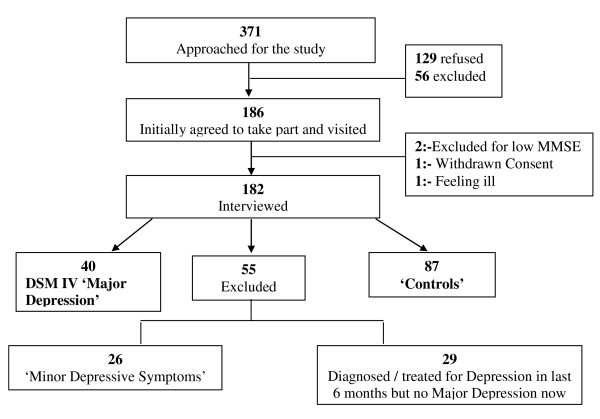
**Study Profile**.

Between March 2002 and September 2003, 186 patients were visited at home and 182 were recruited (Figure 1). Forty of them were found to fulfill the criteria for major depression, and 87 satisfied the control subject criteria. The remaining 55 (29%) met neither set of criteria and were excluded from the analysis. Figure 1 shows the study profile.

Among the cases, 23 (58%) had mild, 16 (40%) had moderate and one had severe major depression according to DSM-IV criteria [9]. Twenty-seven (67.5%) of them had been diagnosed with depression after the stroke but before the interview (6 by psychiatrist, 17 by general practitioner and 4 by stroke physician). Twenty-one (53%) of them were on anti-depressant medication. The mean MADRS score at the time of the interview was 22 (range 18-37) for the depressed group, compared with 1 (range 0-6) for the controls.

### Functional and cognitive status

Demographic characteristics and index stroke features are compared between depressed and control subjects in Table 1. Despite the frequency matching, those in the depressed group were on average two years younger and scored one point less on the 20-point Barthel Index than controls. There were no significant differences between the groups in pre-stroke mobility, continence or modified Rankin scores.

**Table 1:** Demographics, Functional Status and Characteristics of Index Stroke

Variable		Depressed		Controls		
		n		n		p*
Age†	mean ± SD	40	69 ± 11	87	71 ± 10	0.2
Male			58%		61%	0.9
**Functional status**						
Barthel Index†	mean	40	16.5	87	17.6	0.07
(max. 20)	median		17		19	0.003
Frenchay Index	mean	40	11.1	87	20.0	< .001
(max. 66)	median		11		21	< .001
MMSE	mean	40	24.4	86	26.8	< .001
(max. 30)	median		25		27	< .001
Euroquol	mean	40	4.0	87	7.4	< .001
(max. 10)	median		4		8	< .001
**Clinical features of Index Stroke**						
Months Since Stroke.	median (IQR)	40	25 (18-32)	87	26 (21-33)	0.1
**OCSP subtype**		37		82		
TACS		3	8%	4	5%	
PACS		21	57%	46	56%	
LACS		8	22%	18	22%	
POCS		5	13%	14	17%	0.9

Means: One-way ANOVA, Medians: Mann Whitney, Proportions: Chi squared

† **Variables used for frequency matching**

SD: standard deviation, IQR: inter-quartile range.

TACS: total anterior circulation syndrome, PACS: partial anterior circulation syndrome,

LACS: lacunar syndrome, POCS: posterior circulation syndrome

Although differences in basic ADL ability were small, depressed subjects were much less likely to participate in outdoor or social activities (FAI) and were much more aware of limitations in their social participation. Nearly 90% answered "yes" to questions such as "does your health stop you getting around?" and "does your health limit your work and leisure activities?", compared with 44% and 33% of controls (p < 0.0001).

Depressed subjects had significantly more cognitive impairment, with mean MMSE score over 2 points less than controls. Amongst the components of MMSE, cases had significantly lower scores for orientation and recall. As expected, scores on the Euroqol self-rated quality-of-life index were significantly lower in the depressed group.

### Characteristics of the index stroke

There were no significant differences between the groups in the time since the index stroke, distribution of OCSP subtypes (Table 1) or in the side or location of the acute stroke lesion. Only 2 (2%) patients had primary intracerebral haemorrhage and 22 (17%) had posterior circulation strokes, with no difference between groups.

### Socio-economic factors

No significant associations were found between depression and any of the socio-economic factors assessed, including marital status, living alone, educational level, former occupation, recent negative life events and expressed financial worries.

### Vascular and biochemical factors

Table 2 compares vascular and biochemical factors between cases and controls (univariate analysis).

**Table 2:** Cardiovascular Factors

Variable		Depressed (n = 40)		Controls (n = 87)	OR (95% CI)*	p
**Past medical history**	**n**	**%**	**n**	**%**		
Prior Stroke/TIA	16	40%	16	18%	3.0 (1.2 - 7.4)	0.015
Hypertension	30	75%	47	54%	2.6 (1.0 - 6.4)	0.03
Diabetes	5	13%	14	16%	0.7	0.8
Atrial Fibrillation	8	20%	14	16%	1.3	0.6
Myocardial Infarction	11	28%	23	26%	1.1	1.0
Angina	13	33%	21	24%	1.5	0.4
Peripheral Arterial Disease	9	23%	5	6%	4.8 (1.3 - 18)	0.01
Smoker/Ex-smoker	33	82%	55	63%	2.7 (1.0 - 7.7)	0.038
	n	Mean ± SD	n	Mean ± SD		
Alcohol (units/week)	40	10.2 ± 4.8	87	8.5 ± 4.5		0.6
**Blood Pressure (mmHg)**						
Systolic	40	156 ± 22	87	147 ± 19		0.025
Diastolic	40	85 ± 12	87	80 ± 12		0.029
Mean Arterial Pressure	40	109 ± 14	87	102 ± 13		0.014
Pulse Pressure	40	71 ± 17	87	67 ± 16		0.22
**Biochemistry**						
Uric Acid	40	0.39 ± 0.14	87	0.4 ± 0.2		0.8
Total Cholesterol mmol/l	40	5.2 ± 1.1	87	5.1 ± 1.1		0.5
LDL-C	40	2.8 ± 1	87	2.8 ± 0.8		0.7
HDL-C	40	1.4 ± 0.4	87	1.3 ± 0.3		0.15
		Geometric Mean (95% CI)		Geometric Mean (95% CI)		
CRP†	37	5 (3.8-6.6)	76	4 (3.5-4.8)		0.2
Triglyceride†	37	1.9 (1.6-2.3)	82	2.0 (1.8-2.3)		0.8
Platelet Count†	37	267 (240-298)	77	246 (233-259)		0.12
Serum Homocysteine†	37	16.5 (14.5-18.8)	72	14.2 (13.1-15.4)		0.046
Serum Folic Acid†	37	4.7 (3.9-5.6)	73	5.9 (5.2-6.6)		0.03

OR: Odds ratio for depressed vs.control; SD: Standard Deviation;

LDL-C: low density lipoprotein cholesterol; HDL-C: high density lipoprotein cholesterol

*95% CI: 95% Confidence Interval. Only shown where p < 0.1.

†Logarithmic transform used (geometric means tested for significance).

Those with depression were more likely to have a history of hypertension or PAD, or to have suffered a stroke or TIA before their index event. Systolic, diastolic and mean arterial pressures were significantly higher in cases than controls, but patients in both groups were taking a median of two prescribed anti-hypertensive drugs. On the other hand there were no significant differences in the prevalence of ischaemic heart disease or atrial fibrillation between the groups, or in use of antiplatelet or anticoagulant drugs. Depressed patients were more likely to be current or ex-smokers, but self-reported alcohol consumption did not differ between the groups.

Levels of total serum cholesterol, triglyceride, HDL and LDL cholesterol were also similar, and no differences were detected in serum CRP, electrolytes, urea, creatinine, uric acid, albumin, bilirubin, alkaline phosphatase or alanine aminotransferase between cases and controls.

The (geometric) mean homocysteine level was significantly higher, and folate levels correspondingly lower, in the depressed group although differences were modest (Table 2). Serum vitamin B12 levels were only measured in those with serum homocysteine >15 μmol/L, to rule out subclinical deficiency. No instances were found.

### Neuro-imaging findings

Index CT scans were available for analysis for 33 (83%) of the depressed subjects and 70 (80%) of the controls. Fifteen patients were not scanned in Gateshead during the acute phase of their stroke, 5 scans could not be traced and 4 had MRIs, which were not used for this analysis. There were no significant differences in demographic characteristics or vascular risk factors in those with and without available index scans. Focal lesions (recent or established) were seen on 64 (62%) of all scans: 61 ischaemic infarcts, one haemorrhagic infarct and 2 primary haemorrhages. Other abnormalities were identified on 14 (14%) scans; mainly atrophy and diffuse WMCs.

There was moderate agreement (ICC/Kappa = 0.44-0.60) among all raters on most items. Agreement was good for rating central atrophy (ICC = 0.83), but poor for identifying lacunar infarcts (ICC = 0.33) and 'WMCs' within the basal ganglia (ICC = 0.25).

Using the final consensus ratings, the index stroke scans of depressed subjects were more likely to be abnormal (91% vs.71%, OR = 4.0), more likely to show discrete lesions, which were more likely to be small (< 1.5 cm) and/or subcortical. Where acute lesions were identified, however, there were no significant differences in their distribution between left and right hemispheres and brainstem (Table 3).

**Table 3:** Univariate analysis of CT abnormalities between depressed and controls

Variable	Depressed (n = 33)		Controls (n = 70)		OR (95% C.I)
	n	%	n	%	or p-value
**Definite CT Abnormality present**					
**Yes**	30	91%	50	71%	4
**No**	3	9%	20	29%	(1 - 18)
**Discrete Lesion seen** **(acute or chronic)**					
**Yes**	26	79%	38	54%	3.1
**No**	7	21%	32	46%	(1.1- 9.2)
**Size of the Largest Lesion** **(acute or chronic)**					
**< 1.5 cms**	12	46%	8	21%	3.2
**≥ 1.5 cms**	14	54%	30	79%	(1.0 - 11.2)
**Side of the Lesion** **(acute or chronic)**					
**No Lesion**	7	21%	32	46%	
**Right**	14	42%	18	26%	P = 0.08
**Left**	9	27%	12	17%	
**Both Sides**	3	9%	8	11%	
**Location of the Largest Lesion (acute or chronic):**					
**Subcortical (including brainstem)**	16	49%	20	29%	
**Cortical**	10	30%	18	26%	p = 0.04
**No Lesion**	7	21%	32	46%	
**Cortical Atrophy:**					
**Mild/Moderate**	15	46%	30	43%	1.1
**None**	18	54%	40	57%	P = 0.8
**Central Atrophy:**					
**Mild/Moderate**	13	39%	26	37%	1.1
**None**	20	61%	44	63%	P = 0.8
**Lacunar Infarcts present**					
**Yes**	15	46%	20	29%	2.1
**No**	18	54%	50	71%	P = 0.1
**White Matter Changes**	**n**	**Geometric Mean**	**n**	**Geometric Mean**	**p-value**
Overall WMC score*	33	0.55	70	0.28	0.013
Basal Ganglia*		0.28		0.16	0.019
Frontal*		0.57		0.30	0.043
Parieto-occipital *		0.36		0.24	0.2

OR: Odds ratio for depressed vs.control;

95% CI: 95% confidence interval. Only shown where p < 0.1.

* Logarithmic transform used (geometric means tested for significance).

Overall scores for diffuse white matter changes were significantly higher in the depressed group than the controls. Frontal and basal ganglia WMC scores were higher in depressed patients, but within the parieto-occipital region differences did not reach the 5% significance level. We found no significant increase in lacunar infarcts in depressed patients and no correlation at all between depression and the presence or extent of atrophy (Table 3).

### Multivariate analysis

Table 4A and 4B show multivariate logistic regression analysis with presence/absence of depression as dependent variable.

**Table 4:** Multivariate Analysis of Factors Predicting Depression

	4A (39 cases, 86 controls)				4B: (CT results) (32 cases, 69 controls)			
	*β*	Adjusted OR	95% CI	p	*β*	Adjusted OR	95% CI	p
**Matched Variables**								
Age	-0.03	0.97	0.93 - 1.02	0.2	-0.02	0.98	0.93 - 1.03	0.5
Total BI	0.03	0.97	0.84 - 1.11	0.5	-0.1	0.91	0.78 - 1.1	0.2
**'Basic Model' Variables**								
Mean Arterial Pressure	0.05	1.05	1.01 - 1.09	0.016	0.05	1.06	1.01 - 1.1	0.016
Total Number of Strokes	0.67	2.0	1.2 - 3.2	0.033	0.59	1.8	1.1 - 3.0	0.024
PAD	1.7	5.4	1.4 - 20.8	0.014	1.5	4.4	0.99 - 19.5	0.052
Total MMSE	-0.3	0.74	0.63 - 0.88	< 0.001	-0.2	0.80	0.67 - 0.96	0.015
**Folate/Homocysteine**								
Serum Folate*	-0.45	0.64	0.24 - 1.66	0.4	-1.12	0.33	0.1 - 1.05	0.06
Serum Homocysteine*	1.03	2.8	0.69 - 11.5	0.15	1.73	5.6	1.04 - 30.3	0.04
Serum Homocysteine* omitting PAD from model	1.25	3.5	1.0 - 12.7	0.055	2.01	7.5	1.5 - 36.7	0.013
**Other Vascular RFs**								
Diabetes	-1.7	0.18	0.03 - 0.98	0.047	-2.8	0.06	0.01 - 0.66	0.02
AF	0.72	2.06	0.57 - 7.41	0.3	1.0	2.7	0.73 - 10.0	0.14
IHD	0.05	1.05	0.40 - 2.78	0.9	0.2	1.2	0.4 - 3.7	0.7
Smoking History	0.46	1.58	0.73 - 3.46	0.2	0.36	1.4	0.62 - 3.3	0.4
**CT Scan Findings**								
Any abnormality visible					1.05	2.9	1.2 - 6.8	0.017
Discrete lesion visible					1.03	2.8	0.9 - 8.7	0.08
Total No. of lesions					0.39	1.5	0.71 - 3.1	0.3
Lesion location (subcortical vs. cortical)					1.05	2.9	0.95 - 8.6	0.06
Size of the Largest Lesion					0.25	1.3	0.73 - 2.3	0.4
Lacunar Infarct(s) present					0.28	1.3	0.45 - 3.8	0.6
**White Matter Changes**								
Overall WMC Score*					0.39	1.5	0.97 - 2.2	0.07
Basal Ganglia changes*					0.40	1.5	0.97 - 2.3	0.07
Frontal WMCs*					0.22	1.3	0.89 - 1.8	0.2
Parieto-occipital WMCs*					0.09	1.1	0.77 - 1.6	0.6

OR: Odds ratio for depressed vs. control; CT: computed tomography; BI: Barthel Index,

PAD: peripheral arterial disease, RF: risk factor; AF: atrial fibrillation; IHD: ischaemic heart disease; WMCs: white matter changes (includes diffuse ischaemic changes in basal ganglia)

* Natural logarithmic transform used

Age and Barthel score were included in all models, together with those variables significantly associated with depression in univariate analysis: the total number of clinical stroke events, history of hypertension, PAD and smoking, blood pressure readings and MMSE score. Measures such as FAI and Euroqol were not included, as low scores were more likely to have been consequences than causes of depression.

As expected, residual effects of the matching variables were small and statistically insignificant. Table 4A shows that after multivariate adjustment, the effects of current or former smoking were no longer significant, whereas the other variables remained significant. Smoking was therefore eliminated from the model. In the initial model, 'history of hypertension' was associated with an adjusted OR of 3.4 (95%CI: 1.2-9.5), but once information on current blood pressure readings was added, the effect of 'history of hypertension' became insignificant and it was therefore dropped from the 'final basic model'. Serum folate and homocysteine levels were not included in the basic multivariate model because the loss of blood samples meant that data from 14 controls would have been entirely lost from the analysis.

Thus the most important independent predictors of depression in the basic model were the number of stroke/TIA events (odds doubled for each event), history of PAD (odds increased 5-fold if present), mean arterial pressure and MMSE score at the time of interview.

Other variables were then added into the model individually to test their independent effects (Table 4A). Of the 'conventional' vascular risk factors, only diabetes retained borderline significance, being associated with an 80% reduction in the odds of depression. After adjustment, the effects of serum folate and homocysteine (log-transformed and only available in 112 patients) were no longer significant. Since the strong association between peripheral arterial disease and depression was an unexpected finding, the data were reanalysed omitting PAD from the basic model, whereupon the independent effect of homocysteine became more prominent.

Table 4B shows the same analysis for the 103 patients with CT scans available. Overall odds ratio estimates were very similar, except (by chance) those for serum homocysteine, which were double those in Table 4A. Even after multivariate adjustment, the presence of any index CT abnormality was associated with a nearly 3-fold increase in the odds of depression. The presence of subcortical lesions and high WMC scores, particularly in the basal ganglia, were of borderline significance after adjustment.

There was a strong association between overall WMC score and serum homocysteine (Spearman's rho = 0.34, p = 0.001) and both of these were negatively (and possibly causally) associated with MMSE score (rho = -0.21, p < 0.05 for both variables). Thus the adjusted odds ratios for depression were recalculated, omitting MMSE from the basic model, giving a value of 1.56 (95%CI 1.04-2.34, p = 0.03) for overall WMC score, and 6.4 (95%CI 1.31-31.0, p = 0.02) for serum homocysteine (both log-transformed). Both these associations were further strengthened when 'history of PAD' was also omitted from the model.

As in univariate analysis, the extent of cortical or central atrophy and the presence/absence of lacunar infarcts had no significant independent effects.

## Discussion

Our results clearly indicate that vascular and other biological factors are important in the aetiology of late-phase post-stroke depression. Hypertension and accumulated damage due to previous strokes, especially in subcortical areas, appear to be key factors. On the other hand, some strong risk factors for, or indicators of, large artery atheroma (serum cholesterol, symptomatic coronary disease), or cardioembolism (atrial fibrillation), showed little or no effect.

In contrast to other studies, which have relied on screening tests to identify cases of post stroke depression, we used a strict case definition, based on DSM-IV criteria, backed up by a high score on the MADRS scale, a well-validated, reliable assessment, particularly suited to measuring depression severity [19].

All ratings were made by the same person and our control subjects all had low MADRS scores, ensuring wide separation between the groups. Furthermore, subjects were at least 9 months (average over 2 years) post stroke, eliminating the effect of short term adjustment reactions.

On the other hand, much information on risk factors was retrospective and thus potentially open to recall bias [20]. Nearly all such information was checked from other sources, however, so that only self-reported money worries and negative life events were likely to have been influenced by subjects' mood.

We used matching to eliminate, as far as possible, influences on the occurrence of depression of age, which is complex and nonlinear, and of post-stroke disability, which is likely to overwhelm the effects of other factors [21]. Individual matching proved impractical as cases and controls could not be accurately identified before the interview, so we used group frequency matching for these variables. This was successful, but it meant that we could not investigate the effects of age and physical disability in the analysis.

Brain scans were done many months before the interview for clinical management rather than for study purposes, and were not available for all subjects. Thus unknown structural changes could have taken place during the intervening period, which might have had a bearing on the occurrence of depression.

Finally, simultaneous testing for multiple risk factors increases the risk of identifying chance associations. We therefore gave priority to analysing associations, which formed part of our prior hypothesis of a link between cerebrovascular risk factors and post-stroke depression.

The strong association between a history of peripheral arterial disease and depression, both in univariate analysis and after adjustment, was unexpected. None of the subjects interviewed had obvious ischaemic rest pain or amputations, and no direct measurements of ankle/brachial pressure index were made. Thus classification depended on a history of claudication or previous treatment for PAD. Since associations between reported PAD and risk factors such as diabetes and coronary disease were weak, this classification may have been unreliable and the apparent link between PAD and depression spurious. When PAD was left out of the logistic model, the strength of association of factors such as homocysteine with depression was increased.

The association of serum folate and homocysteine with depression, significant in univariate analysis, was also attenuated by the presence of cognitive impairment, and we found significant associations between low MMSE scores, serum homocysteine and chronic white matter changes on CT scan.

Along with physical disability and stroke severity, cognitive impairment is one of the few factors consistently associated with depression after stroke [21]. The relationship is complex, but impaired cognition may at least partly be a consequence of depression and there is some evidence that effective treatment or spontaneous resolution of depression may have lasting effects on cognition [22,23]. Thus it was appropriate to repeat the logistic regression analysis, omitting the MMSE score from the model, which significantly increased the odds ratios for homocysteine and WMC score.

Thus our data are consistent with a causal link between raised homocysteine levels, white matter changes on CT scans, cognitive impairment and the occurrence of post stroke depression. A possible toxic effect of homocysteine on endothelial function has been proposed, and raised levels seem to be associated with cerebral small vessel disease, particularly that manifesting as diffuse leukoaraiosis rather than as discrete lacunar infarcts [24]. Evidence is emerging that these two subtypes of small vessel disease may differ in their aetiology [25], with atherosclerotic risk factors such as raised cholesterol, coronary disease and diabetes being important in the second type, and only hypertension consistently related to the first.

Our finding that depressed stroke survivors had significantly more diffuse changes in deep white matter and basal ganglia, but no more lacunar infarcts than nondepressed controls, suggests that post-stroke depression may be linked with the diffuse type of small vessel disease but not the discrete lacunar type. Only subcortical lesions seemed to predict depression; the extent of cortical damage, cortical or central atrophy having no apparent effect. Diffuse changes around the basal ganglia showed the strongest association, frontal periventricular changes were of borderline significance, whereas changes in parieto-occipital white matter showed no discernable association with depression. These relationships are strikingly similar to those found by the LADIS Group [26], who investigated the links between depressive symptoms and MRI white matter changes in nondisabled older people.

Our finding that both systolic and diastolic pressures, but not pulse pressure, were significantly associated with post-stroke depression and cognitive impairment, again indicates that potentially causal vascular factors may be acting distal to large arteries. This is consistent with the findings of the Rotterdam Study, which found no associations between measures of large artery stiffness and cognitive decline, after adjustment for other cardiovascular factors [27].

Thus our results seem to support the 'vascular depression hypothesis' [28], which proposed that vascular lesions might lead to depression by disrupting prefrontal circuits or their modulating pathways. Diffuse ischaemic damage due to small vessel disease, especially around the basal ganglia and frontal white matter, would be expected to have a greater effect than multiple discrete infarcts, and an association between such changes and late life depression (in contrast to early onset depression) has been convincingly demonstrated [29].

Homocysteine might also be involved in this process, either by influencing deep white matter changes [30,31], or possibly through a direct effect on neurotransmitters [31]. The relationships between homocysteine, other vascular risk factors, white matter changes, cognitive impairment and depression are complex and may vary according to age and the overall level of risk in the population studied. They are only likely to be disentangled by longitudinal studies and intervention trials [32] and early findings have cast doubt on some of the causal links [33-37]. Our results suggest that the best prospect for testing the 'homocysteine/vascular hypothesis of depression' would be to conduct trials of blood pressure lowering or neurotransmitter modulation and/or folate and B-vitamin supplementation in high risk stroke survivors with diffuse small vessel disease.

## Conclusion

Our results suggest that history of multiple strokes, hypertension, smoking, peripheral vascular disease and visible discrete subcortical lesion or basal ganglia changes on baseline CT brain scans had independent association with major post-stroke depression at the chronic phase. Furthermore, patients with major post-stroke depression had greater degree of cognitive impairment, higher serum homocysteine and lower serum folate levels compared with stroke survivors of similar age and functional ability. Therefore, some risk factors for small vessel cerebro-vascular disease may also be risk factors for clinical depression in the chronic phase after stroke.

## Competing interests

The authors declare that they have no competing interests.

## Authors' contributions

KC and DB made a substantial contribution towards study design, analysis and interpretation of data as well as drafting the manuscript. KC and SF made a substantial contribution towards acquisition of data and SF contributed towards screening process, as KC was blinded from it. All authors read and approved the final manuscript.

## Pre-publication history

The pre-publication history for this paper can be accessed here:

http://www.biomedcentral.com/1471-2377/10/125/prepub
